# Synchronous Colon and Breast Cancers: A Case Report of Multiple Primary Tumors

**DOI:** 10.7759/cureus.24798

**Published:** 2022-05-07

**Authors:** Moneera Y Bin Saleem, Mahdi H Albandar, Jaber A Alfaifi

**Affiliations:** 1 General Surgery, King Abdullah Bin Abdulaziz University Hospital, Princess Nourah Bint Abdulrahman University, Riyadh, SAU; 2 Department of Surgery, King Saud Medical City, Riyadh, SAU

**Keywords:** invasive ductal cell carcinoma, adenocarcinoma, synchronous tumors, breast cancer, colon cancer

## Abstract

Multiple primary malignant tumors (MPMNs) are not rare entities. We report a case of a 50-year-old female who presented with left upper quadrant abdominal pain and GI bleeding. Initial assessment with CT scan revealed a mass originating from the descending colon. Colonoscopy was performed and a fungating partially obstructed mass at the left splenic flexure was detected. Histopathological examination of biopsy was consistent with mucinous adenocarcinoma. The staging CT scan of the chest and pelvis, followed by a mammogram reported a Synchronous breast mass. Core needle biopsy detected an invasive ducal carcinoma. In the multidisciplinary meeting, it was decided to perform the colon procedure first, followed by adjuvant chemotherapy, and then the breast procedure. The patient had an uneventful recovery after both procedures and was sent to the medical oncology department to continue with the treatment.

## Introduction

Multiple primary malignant tumors (MPMNs) comprise two or more tumors with different pathological origins [1]. They can be synchronous or metachronous. Tumors that occur within six months of a previous malignant tumor diagnosis are defined as synchronous neoplasms [2], which are challenging to diagnose, and their management can also be challenging due to inadequate guidelines. In this study, we report a surgical case of multiple primary synchronous tumors, a patient with colon cancer in which a synchronous breast tumor was incidentally detected during the colon tumor staging.

## Case presentation

A 50-year-old postmenopausal female patient presented with a history of left upper quadrant abdominal pain associated with black tarry stool and fresh rectal bleeding that has been present for the last 9 months. She also had experienced nausea, night sweats, and progressive weight loss. Her bowel habits had not changed. Her past medical history included hypertension. Additionally, she had no family history or environmental exposition to risk factors. A head and neck examination showed a right thyroid lobe swelling but no cervical lymphadenopathy. Abdominal examination revealed a palpable tender mass in the left upper quadrant. The digital rectal examination showed melena. Nevertheless, vital signs were within the normal range

Investigation

**Figure 1:**
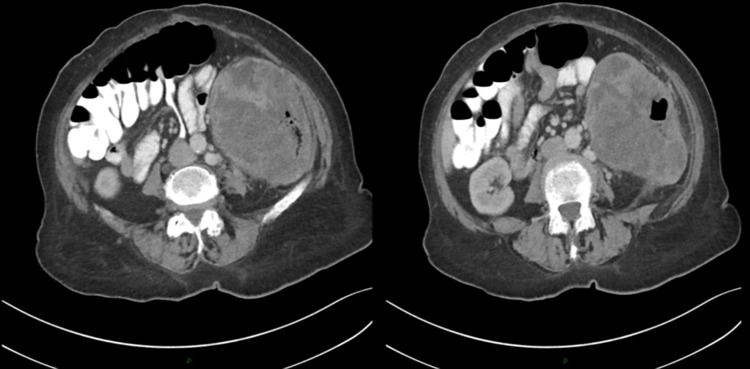
Computed tomography scan showing a large heterogeneous mass originated from the descending colon with surrounding multiple regional lymph nodes and fat stranding

**Figure 2:**
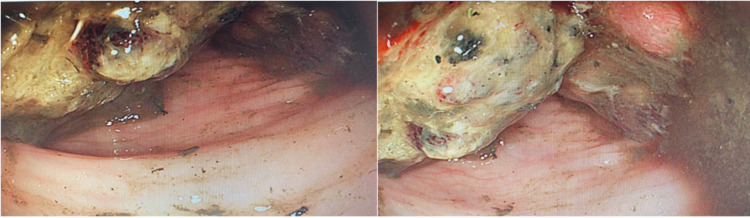
Colonoscopy showing a fungating partially obstructed mass at the left splenic flexure

Laboratory results on presentation revealed anemia (Table 1); thus, a blood transfusion was performed. Tumor markers were elevated (Table 1). Computed tomography (CT) of the abdomen showed a heterogeneous mass measuring 14×12×9cm, which originated from the descending colon with surrounding regional lymph nodes and fat stranding (Figure 1). Colonoscopy was performed, which showed a fungating partially obstructed mass at the left splenic flexure (Figure 2).

**Table 1:** Laboratory results on presentation AFP: Alpha-fetoprotein; CEA: Carcinoembryonic antigen; CA19-9: Carbohydrate antigen 19-9;  CA125: Cancer antigen 125; CA15-3: Cancer antigen 15-3.

Complete blood cell count		
White blood cell	12	4.8–10.8 K/µL
Red blood cell	3.05	4.70–6.10 M/µL
Hemoglobulin	5.4 g/dL	14.0–18.0 g/dL
Hematocrit	20.2	42.0–52.0%
Platelets	587	130–400 K/µL
Complete metabolic panel		
Potassium	2.6	3.5–5.2 mmol/L
Sodium	132	135–148 mmol/L
Chloride	101	100–110 mmol/L
Bicarbonate	26	21–32 mmol/L
Blood urea nitrogen	24	3.0–23.0 mg/dL
Creatinine	1.20	0.80–1.30 mg/dL
Aspartate aminotransferase	36	0–48 U/L
Alanine aminotransferase	33	13–61 U/L
Albumin	3.1	3.4–5 g/dL
Tumor markers		
AFP	4.34	0 - 9
CEA	79.70	0 - 5 ng/mL
CA 19-9	254.90	0 - 35 u/mL
CA125	54.50	0 - 35 u/mL
CA15-3	3.00	0 - 31.3 u/mL

The biopsy was consistent with invasive mucinous adenocarcinoma. The chest and pelvic staging CT scan reported an enlarged right thyroid lobe with multiple hypoattenuation lesions with peripheral rim calcification and a left enhanced breast mass. The patient then underwent mammography and thyroid ultrasonography (US). The mammography reported a speculated round mass at the left upper outer quadrant measuring 1.9×1.7 cm with spicules extended anteriorly and posteriorly. Multiple benign-looking lymph nodes were seen in both axillae. Core needle biopsy of the left breast mass documented a grade 2 invasive ducal carcinoma. Immunohistochemical examination revealed estrogen receptor (ER), progesterone receptor, and human epidermal growth factor receptor 2 (HER2) positive. US thyroid showed bilateral multiple well-defined hyperechoic nodules and increased vascularity with no enlarged lymph nodes. Fine needle aspiration of the thyroid confirmed the diagnosis of Hashimoto thyroiditis.

Treatment

**Figure 3:**
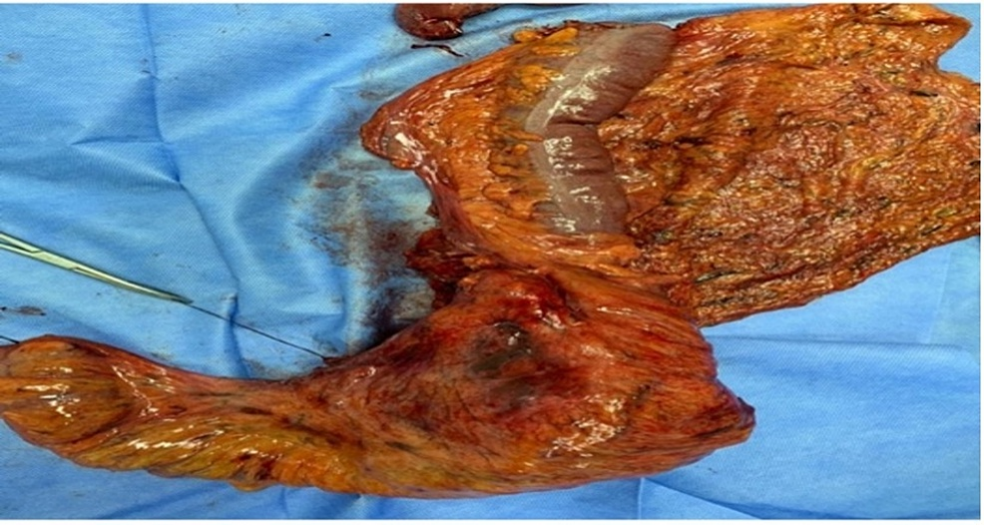
Macroscopic look of colon cancer is eliminated from left colon by extended left hemicolectomy.

The clinical picture of the patient was discussed in a multidisciplinary oncology meeting, wherein the colon procedure was decided to be performed first, followed by adjuvant chemotherapy and breast procedure. The left extended hemicolectomy and colocolonic anastomosis were uneventful (Figure 3). Histopathology examination of the resected specimens revealed an 18cm moderate to well-differentiated mucinous adenocarcinoma, with invasion to the muscularis propria into the subserosal fat. Out of the 19 regional lymph nodes, two were positive for metastasis, making the pathological stage a pT3N1. Anti-Her2 pertuzumab and trastuzumab were started while she was waiting for chemotherapy. After completing four cycles of XELOX, she underwent total mastectomy and sentinel lymph node biopsy (SLNB). No invasion was detected in the SLNB; thus, axillary dissection was not performed. Histopathology examination revealed a 4cm residual invasive ductal carcinoma not otherwise specified and a ductal carcinoma in situ constituting 5%. All surgical resected margins were tumor-free.

Outcome and follow up

The patient had an uneventful recovery after both procedures. The postoperative plan is to continue with clinical and radiological follow-up as recommended by the multidisciplinary team (MDT) and to continue the Anti-Her2 therapy with the medical oncology department.

## Discussion

MPMNs comprise two or more tumors with different pathological origins [1]. Its incidence increased with technical advances in cancer diagnosis, which is estimated between 0.73% and 11% [3]. Most MPMN cases appear metachronous with a prevalence of 17% [4]. By contrast, synchronous tumors are rare and often attributed to known and unknown genetic predispositions [5].

The following reasons confirmed the diagnosis of synchronous MPMN in the present case report. First, the patient had two tumors, invasive mucinous colon adenocarcinoma, and invasive ducal breast carcinoma. Second, the interval between the occurrences is <6 months. Third, both tumors were different in pathological type, excluding a metastatic origin for one of them, which meets the inclusion criteria of Warren and Gates to be defined as an MPMN [2].

The synchronous occurrence of breast and colon cancers is an infrequent clinical entity with a 3.85% incidence [6]. Well-studied inherited mutations can predispose individuals and their families to a variety of multiple malignancies, such as mismatch-repair genes in Lynch syndromes [7], p53 (Li-Fraumeni syndrome) [8], and familial adenomatous polyposis. However, breast cancer is not included as a subtype of hereditary nonpolyposis colorectal cancer (CRC), and our patient does not fulfill The Amsterdam II Criteria for Lynch syndrome diagnosis.

Our patient also presented a thyroid nodule; fortunately, it was a benign pathology. However, any thyroid nodule presented with a breast mass should be carefully investigated. Compared with the normal population, women with thyroid malignancy have a higher risk of developing a second tumor, wherein the breast is the most frequent site [9]. Moreover, some genetic syndromes, such as the Cowden syndrome, are associated with thyroid and breast cancers [10]. Thyroid nodules in patients with CRC can also present a challenging diagnostic and management problem. In such cases, immunohistochemical staining for cytokeratins (CK) 7 and 20 can help distinguish a primary thyroid malignancy from metastatic colon adenocarcinoma [11].

Principal risk factors known for MPMNs are alcohol abuse, tobacco, carcinogen exposure, or a personal or family history of cancer [12], which we excluded. Thus, in our patient’s case, the patient likely developed sporadic synchronous primary tumors that were unrelated to any familial predisposition or known risk factors.

In our MDT meeting, the colon procedure was decided to be performed first due to the patient’s clinical presentation of bleeding and then followed by adjuvant chemotherapy and breast procedure. In such cases, the role of the MDT is invaluable to determine the treatment plan and individualize it for each patient.

## Conclusions

Improved cancer care and the technical advancement in cancer diagnosis have led to a decreased incidence of missing MPMNs. Synchronous colon and breast cancer are rare, especially in the absence of genetic susceptibility. The treatment plan should be individualized for each patient due to inadequate definitive guidelines for the management of such cases through a complete preoperative evaluation and MDT meeting to provide the best possible treatment for the patient.
